# Exploration of a Novel Electric-Fuse Device with a Simple Structure of Ni Metal on a SiO_2_ Dielectric for Electrostatic Discharge Protection under a Human Body Model

**DOI:** 10.3390/mi15091163

**Published:** 2024-09-19

**Authors:** He Guan, Jiaying Li, Yangchao Chen, Yongchuan Tang, Yunshuo Li

**Affiliations:** 1 School of Microelectronics, Northwestern Polytechnical University, Xi’an 710072, China; 2020302234@mail.nwpu.edu.cn (J.L.); chenyangchao@mail.nwpu.edu.cn (Y.C.); tangyongchuan@nwpu.edu.cn (Y.T.); 2016301981@mail.nwpu.edu.cn (Y.L.); 2 Chongqing Innovation Center, Northwestern Polytechnical University, Chongqing 401135, China

**Keywords:** electrostatic discharge, electric fuse, transmission line pulse

## Abstract

On-chip electrostatic discharge (ESD) protection poses a challenge in the chip fabrication process. In this study, a novel electric fuse (E-fuse) device featuring a simple structure of Ni metal on a SiO_2_ dielectric for ESD protection was proposed, and the physical mechanism of its operation was investigated in detail. Experimental evaluations, utilizing transmission line pulse (TLP) testing and fusing performance analyses, reveal that the E-fuse, constructed with a Ni metal layer measuring 5 μm in width, 100 μm in length, and 5 nm in thickness, achieved a significant ESD protection voltage of 251 V (*V_HBM_*) and demonstrates low-voltage fusing at a bias voltage of 7 V. Compared to traditional ESD protection devices, the E-fuse boasts a smaller size and removability. To assess fusing performance, devices of varying sizes were tested using a fusing lifetime model. This study supports both theoretical and empirical evidence, enabling the adoption of cost-effective, straightforward E-fuse devices for ESD protection.

## 1. Introduction

As chip processes are reduced, they become more susceptible to ESD. ESD can occur throughout the entire chip manufacturing process, including during the preparation, packaging, and shipping stages of integrated circuits (ICs). This can damage sensitive components and interfere with their operation, resulting in reduced chip performance or even failure. Therefore, ESD protection is receiving increasing attention as an important aspect of chip reliability [1,2,3].

ESD protection can be divided into off-chip and on-chip ESD protection according to different protection positions. Off-chip ESD protection devices are mainly Ceramic Capacitors, Zener Diodes, Schottky Diodes, Multi-Layer Varistor (MLV) and Transient Voltage Suppressors (TVSs) [4,5,6,7,8], which are generally placed in the peripheral circuits of the chip. However, off-chip ESD protection devices are difficult to integrate and cannot meet the miniaturization requirements. On-chip ESD protection devices are mainly diodes, Gate-Grounded NMOS (GG-NMOS), and Silicon Controlled Rectifier (SCR) [9,10,11]. Compared to off-chip ESD protection circuits, on-chip ESD protection circuits can directly and significantly enhance ESD protection, save board space, reduce system costs, and decrease design and wiring complexity. However, this inevitably introduces parasitic parameters and increases layout complexity. In 2014, Kuhn et al. proposed the use of E-fuse devices for ESD protection, providing new ideas for on-chip ESD protection [12]. Compared with traditional on-chip ESD protection devices, E-fuse protection devices have the advantages of small size, low parasitic parameters, and removability, making them very suitable for one-time electrostatic protection in the chip manufacturing process [13].

**Figure 1:**
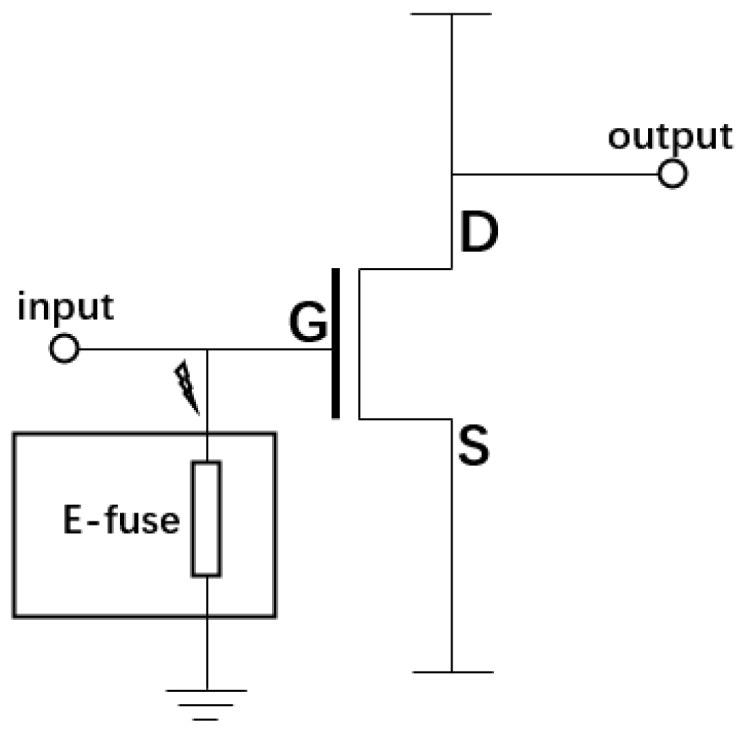
The circuit diagram of the E-fuse device applied in MOSFET.

Based on this background, this paper presents an improved E-fuse ESD protection device with a simpler structure. The device includes a nano-scale Ni metal layer lithographically covered on the SiO_2_ dielectric layer, which is very easy to integrate onto the surface of any semiconductor device through a semiconductor device process to realize on-chip ESD protection. As an illustration example, as shown in Figure 1, the E-fuse device could be connected to the gate of the MOSFET devices for ESD protection. When high-voltage static electricity is generated during the operation, the E-fuse device is used to discharge the accidental static electricity in order to protect the gate of the MOSFET device from ESD damage. It is noted that, when a continuous low-voltage is added, the E-fuse device will be fused off by electromigration and will not affect the working performance of the MOSFET. This means that the protection device can be easily removed, reducing the impact on the internal circuitry.

The specific studies in this paper are as follows. Firstly, the design structure and manufacturing process of the E-fuse device are introduced, and the working physical mechanism is investigated in detail. Among the various types of electrostatic discharges, human body electrostatic damage is a significant component of chip electrostatic damage, posing a significant threat to semiconductor devices [14]. Therefore, the TLP test, which is considered to be the typical evaluation measurement of an ESD human body model (HBM), is applied to the device to characterize the ESD performance. In addition, a fusing experimental test was conducted on the device, the fusing lifetime model was discussed based on devices with various sizes, and a detailed analysis of the results was performed. The test results show that the prepared E-fuse device, with a length of 100 μm, a width of 5 μm, and a thickness of 5 nm, had an ESD protection voltage of 251 V and achieved low-voltage fusing at a bias voltage of 7 V.

## 2. Device Preparation

### 2.1. Structural Design

**Figure 2:**
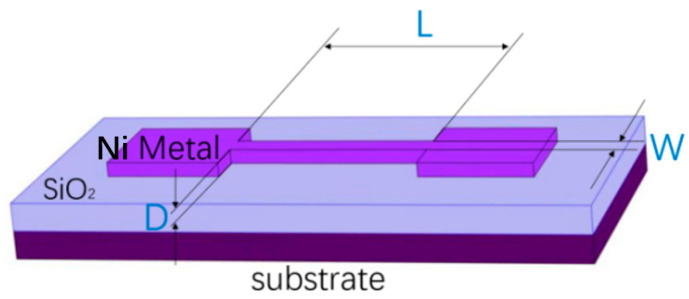
Structural diagram of the E-fuse device.

The E-fuse protection device was designed as shown in Figure 2. Based on the substrate (Si, as the most common semiconductor, was selected in this paper), a SiO_2_ dielectric layer with 200 nm thickness is deposited. Above the dielectric, Ni metal was chosen for use as the E-fuse part because of its high strength, corrosion resistance, heat resistance, and good conductivity [15]. The length, width, and thickness of the E-fuse are represented as *L*, *W*, and *D*, respectively. The rectangular parts on the left and right sides of the thin E-fuse are metal electrodes for convenient testing. Obviously, E-fuse devices prepared with pure Ni metal are much simpler and easier to integrate compared to conventional ESD protection devices.

### 2.2. Preparation and Characterization

The device is fabricated using nanoscale semiconductor technology with the following steps: (1) Cleaning of wafers [16]. The wafer samples are placed in a beaker with acetone and then isopropyl alcohol (IPA) is added. All organic solvents are subjected to an ultrasonic water bath to remove organic contaminants from the sample surface. The samples are then rinsed with deionized water and dried with a nitrogen gun to complete the cleaning procedure [17]. (2) Deposition of polysilicon [18]. A 200 nm silicon dioxide dielectric layer is deposited on the wafer using a low-pressure chemical vapor deposition (LPCVD) process [19]. (3) Preparation of E-fuse devices [20,21,22].

**Figure 3:**
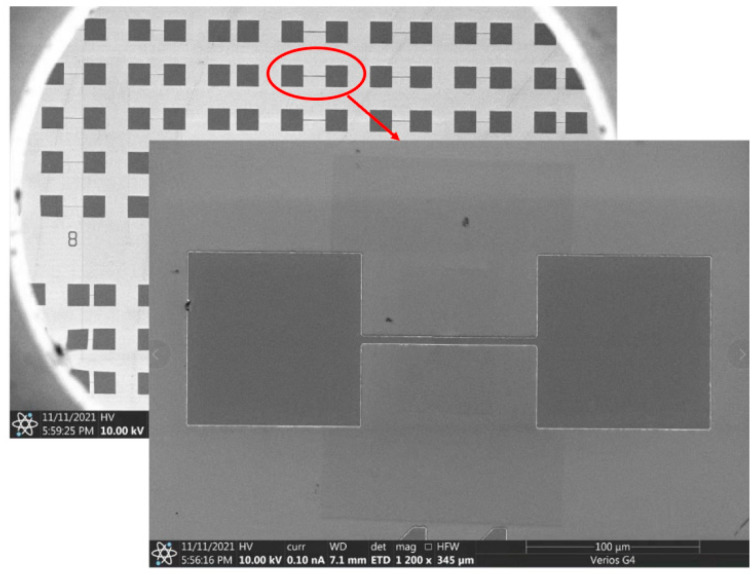
E-fuse device under SEM test.

The preparation process follows the order of adhesive coating, electron beam lithography (EBL), development, and metal thermal vapor deposition. First, the photoresist is uniformly spin-coated on the sample surfaces. Next, EBL is performed. The sample is placed in the photolithography equipment and the mask is aligned. After the pattern on the mask has been aligned, the sample is brought into contact with the mask, the photoresist is exposed to UV light, and the sample is immersed in deionized water to remove the conductive adhesive. Development is then performed. The sample is immersed in the developer (4-Methyl-2-pentanone and IPA), and then washed and dried with isopropanol and N_2_. O_2_ plasma is used to remove residual rubber backing from the sample surface. Finally, metal thermal evaporation is performed. Metals are deposited using an electron beam metal evaporator. Then, the samples are placed in acetone and sonicated, followed by immersion in a mixture of acetone and IPA to complete the metal stripping. The sample is re-immersed in deionized water and an ultrasonic water bath, and then dried with N_2_. The image of the prepared device as viewed through a scanning electron microscope (SEM) is shown in Figure 3.

**Figure 4:**
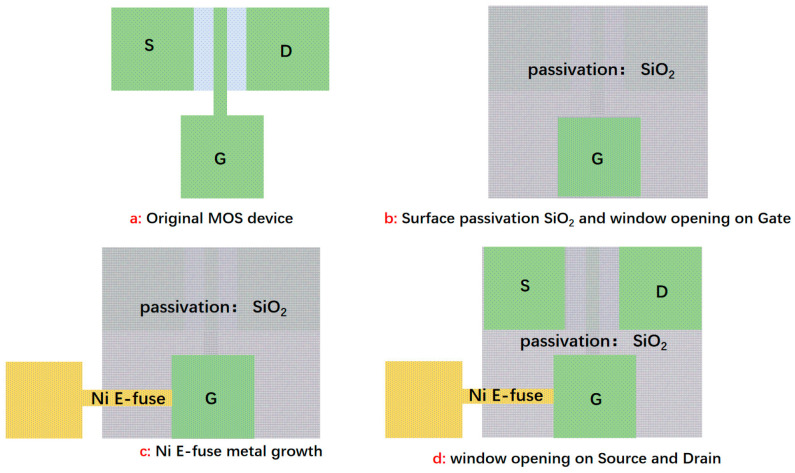
The illustration and diagram of the process and layout of the E-fuse device applied in MOSFET, where G, S, and D denote the gate, source, and drain of the MOSFET, respectively.

In practice, the process of this device is also very simple. For the application shown in Figure 3, as the example, the layout diagram is as shown in Figure 4. It requires three simple steps for surface passivation with SiO_2_ and window opening on the gate, Ni E-fuse metal deposition, and window opening on source and drain.

## 3. Testing and Analysis

### 3.1. TLP Test

**Figure 5:**
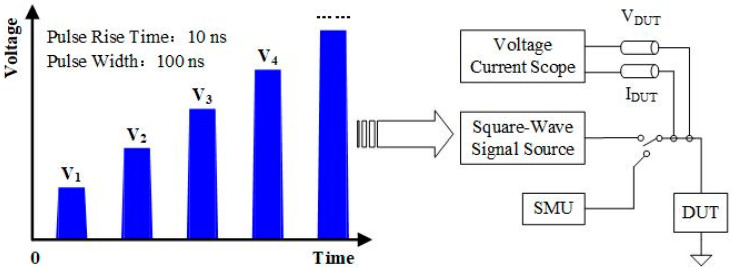
TLP block diagram.

**Figure 6:**
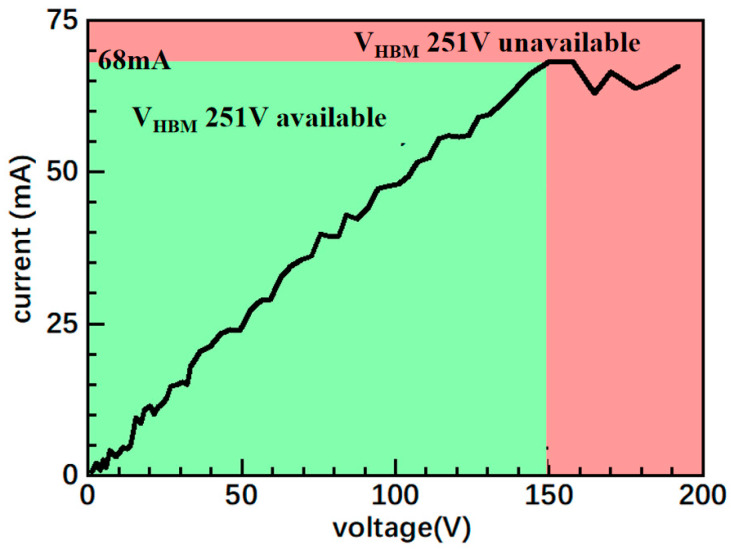
E-fuse device TLP test results.

The TLP test was applied on a sample with a Ni E-fuse metal length *L* of 100 μm, width *W* of 5 μm, and thickness *D* of 5 nm to simulate the HBM. The TLP test procedure is shown in Figure 5. By gradually applying increasing voltage pulses to both ends of the device under test (DUT) and recording the voltage across the DUT and the current flowing through it under each pulse, the TLP test curve of the DUT can be obtained. Typically, the rise time of the voltage pulse applied for the TLP test is 10 ns and the pulse width is 100 ns [23,24]. Figure 6 illustrates the TLP test results; when the pulse voltage across the device is less than 149 V and the current is less than 68 mA, the TLP test curve of the device has an approximately constant slope, which is consistent with the normal leakage current characteristics of E-fuse devices. At this stage, the E-fuse resistance *R*_on_ can be calculated to be approximately 2191 Ω. When the voltage rises above 149 V and the current exceeds 68 mA, the resistance of the device rises sharply, indicating that the device is damaged by the current generated during the TLP test. During this period, the secondary thermal escape current (*I*_*t*2_) can reach as high as 68 mA. Since the E-fuse resistance is significant, the protection voltage calculation equation is expanded so that Equation (1) [25] can be obtained, in which 1500 represents the equivalent resistance of the human body in the HBM. The final protection voltage *V_HBM_* of the device was calculated to be 251 V, which is higher than that of similar devices [26,27]. To further enhance the *V_HBM_* values, the current density can be reduced by increasing the size of the E-fuse device, which results in a larger *I_t_*_2_ and *V_HBM_*.

(1) $$V_{HBM}=(1500+R_{on})\times I_{t2}$$

### 3.2. Fusing Test

After completing the TLP test, a fusing test was performed to demonstrate that the device could be successfully removed. Unlike the TLP test, which applies a continuously increasing pulse voltage, the fusing test applies a constant voltage across the device terminals until the device fails, and the corresponding fusing time is recorded. The fusing mechanism of the E-fuse device can be explained by electromigration, and the corresponding mechanism is described as follows. Under the continuous application of a certain voltage, the E-fuse will fuse due to electromigration. The physical theory of electromigration can be explained as follows. Under the influence of the current, metal atoms are displaced, leading to the formation of cavities in some areas of the metal. Similarly, other areas develop crystal hills due to the accumulation of metal atoms piled up on the surface or within the metal [28,29]. The electrons in the metal gain high speed under the influence of the electric current, and these high-speed electrons collide with the metal atoms. Through these collisions, the metal atoms gain momentum, leading to their acceleration. This movement of metal atoms, driven by what is known as electron wind force, is also influenced by the electrostatic field. However, when the current density in the circuit is high, electrons diffuse toward the anode under the current density’s action. The resulting electron wind force becomes greater than the electrostatic field force. Therefore, under the combined influence of the electron wind force and the electrostatic field, metal atoms undergo directional diffusion from the cathode to the anode. The accumulation of the directional motion of metal atoms at the microscopic level results in metal fusion, causing the current to drop to 0 [30,31,32].

Electromigration is a complex process, involving several physical and chemical factors. Therefore, it is necessary to comprehensively consider various factors in practical applications to evaluate the performance and reliability of the E-fuse. To analyze the effect of E-fuse sizes and metal materials on the fusing performance of the device, we manufactured different E-fuses with various lengths and widths, and applied different voltages to both ends of the device for fusing tests.. The fusing test results, such as fusing time and fusing voltage, are shown in Table 1. It is obvious that at higher DC voltages, E-fuse devices of the same size have a shorter fusing time. Therefore, in specific applications, the fusing time can be changed by adjusting the size of the device to meet different application scenarios.

Here, Black’s equation [33] is used as a model for evaluating the fusing performance of E-fuse devices at different sizes, as shown in Equation (2).

(2) $$MTTF=A\times J^{-n}\times Exp\left(\frac{E_{a}}{k_{b}\times T}\right)$$

where *MTTF* refers to the electromigration lifetime, specifically the mean time to failure; *A* and n are constants related to the material, conductor geometry, and current distribution; *J* refers to the current density when the device fuses; *Ea* represents the activation energy of electromigration, which is a characteristic of the material and indicates the energy barrier that needs to be overcome during the electromigration process; *k_b_* is Boltzmann’s constant; and *T* refers to the absolute temperature.

The equation indicates that a higher current density will increase the collision frequency between electrons and metal atoms, thereby accelerating the electromigration process. Consequently, the E-fuse device may be more prone to fusing off under higher current densities, meaning that *MTTF* is inversely proportional to *J*. In Table 1, the fusing current density *J* can be calculated through the E-fuse size and fusing voltage. It is found that the fusing time of devices with different sizes is inversely proportional to their current density. Therefore, the fusing time trend of the devices is consistent with the *MTTF* indicated in the model formula; that is, the larger the *MTTF* is, the longer the fusing time is. However, it is assumed here that the temperature is constant. In practice, according to Joule’s law, the current flowing through the E-fuse device will inevitably generate heat, which will have some effect on the *MTTF*. Therefore, in subsequent work, the effect of temperature on *MTTF* will also be considered as much as possible.

**Figure 7:**
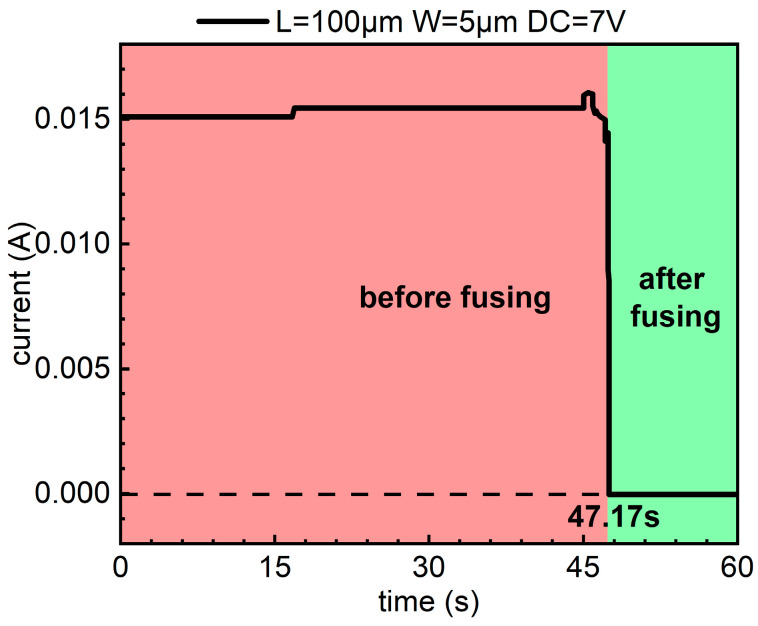
Fusing test results of the device.

The device with a Ni metal length of 100 μm, a width of 5 μm, and a thickness of 5 nm, which was selected to illustrate ESD test above, presents a fusing performance as shown in Figure 7. It is found that the device is blown under a 7 V voltage in approximately 47.17 s. It is indicated that the devices exhibit low-voltage fusibility. The specific application of the fusing phenomenon is that after the circuit assembly has been completed, the fuse element is blown by an externally applied low DC voltage to finalize the circuit configuration. Disabling or triggering the ESD circuit in this manner will not cause any damage to the protected device. This E-fuse device is a competitive solution for low-cost and reliable on-chip ESD protection requirements.

Further tests show that the E-fuse device generates a capacitance of about 40 pF after fusing, primarily due to the parasitic effects of preparing the device directly on the Si substrate for testing purposes. Although this capacitance is equivalent to that of a conventional DC isolation capacitor, its self-resonance frequency is high, which affects high-frequency signals but has less of an impact on most low-frequency analog and digital circuits. In practice, E-fuse devices can be fabricated on layers other than the substrate to reduce the impact of parasitic effects. We will continue to improve the device fabrication process to reduce the parasitic capacitance generated after fusing, thereby expanding the application areas of E-fuse protection devices. Furthermore, we will continue our research on improving the E-fuse ESD structure by optimizing its size, metal composition, and fabrication process, aiming to achieve lower resistance and higher voltage ratings for future high-level HBM standard applications.

## 4. Conclusions

In conclusion, this study has successfully demonstrated the potential of an innovative SiO_2_-Ni electrical fuse device as an effective on-chip ESD protection solution. Through TLP tests and fusing performance evaluations, our findings reveal that the fabricated E-fuse device, featuring a Ni metal of 100 μm length, 5 μm width, and 5 nm thickness, exhibits a remarkable protection voltage of 251 V while achieving low-voltage fusing at only 7 V. Furthermore, an analysis of devices with varying dimensions is applied to develop a fusing lifetime model, enhancing the understanding of the device’s fusing performance. This work primarily validates the feasibility of using cost-effective and simple-design Ni metal E-fuse devices for ESD protection applications, and presents their theoretical basis, thereby offering new insights and potential avenues for advancements in chip protection technology. We will continually improve the protection voltage and microsecond-scale fusing time in our upcoming study to make them more readily available for various applications.

## Figures and Tables

**Table 1:** Fusing test results with various e-fuse sizes and metal materials.

D/[nm]	L/[μm]	W/[μm]	Fusing Voltage/[V]	Fusing Time/[s]	Fusing Resistance/[Ω]	Fusing J/[mA/μm^2^]
50	100	5	7	47.17	3200	61.25
50	30	3	4.5	11.22	1600	84.38
50	30	3	5	10.76	1600	104.17
50	25	5	5	3.57	800	125
50	25	5	6	2.1	800	180

## Data Availability

Data are contained within the article.
